# Two Distinct Cdc2 Pools Regulate Cell Cycle Progression and the DNA Damage Response in the Fission Yeast *S*.*pombe*

**DOI:** 10.1371/journal.pone.0130748

**Published:** 2015-07-01

**Authors:** Thomas Caspari, Victoria Hilditch

**Affiliations:** Genome Biology Group, School of Medical Sciences, Bangor University, Bangor, LL57 2UW, Wales, United Kingdom; Florida State University, UNITED STATES

## Abstract

The activity of Cdc2 (CDK1) kinase, which coordinates cell cycle progression and DNA break repair, is blocked upon its phosphorylation at tyrosine 15 (Y15) by Wee1 kinase in the presence of DNA damage. How Cdc2 can support DNA repair whilst being inactivated by the DNA damage checkpoint remains to be explained. Human CDK1 is phosphorylated by Myt1 kinase at threonine 14 (T14) close to its ATP binding site before being modified at threonine 161 (T167^Sp^) in its T-loop by the CDK-activating kinase (CAK). While modification of T161 promotes association with the cyclin partner, phosphorylation of T14 inhibits the CDK1-cyclin complex. This inhibition is further enforced by the modification of Y15 by Wee1 in the presence of DNA lesions. In *S*.*pombe*, the dominant inhibition of Cdc2 is provided by the phosphorylation of Y15 and only a small amount of Cdc2 is modified at T14 when cells are in S phase. Unlike human cells, both inhibitory modifications are executed by Wee1. Using the novel IEFPT technology, which combines isoelectric focusing (IEF) with Phos-tag SDS electrophoresis (PT), we report here that *S*.*pombe* Cdc2 kinase exists in seven forms. While five forms are phosphorylated, two species are not. Four phospho-forms associate with cyclin B (Cdc13) of which only two are modified at Y15 by Wee1. Interestingly, only one Y15-modified species carries also the T14 modification. The fifth phospho-form has a low affinity for cyclin B and is neither Y15 nor T14 modified. The two unphosphorylated forms may contribute directly to the DNA damage response as only they associate with the DNA damage checkpoint kinase Chk1. Interestingly, cyclin B is also present in the unphosphorylated pool. We also show that the G146D mutation in Cdc2.1w, which renders Cdc2 insensitive to Wee1 inhibition, is aberrantly modified in a Wee1-dependent manner. In conclusion, our work adds support to the idea that two distinct Cdc2 pools regulate cell cycle progression and the response to DNA damage.

## Introduction

**Fig 1:**
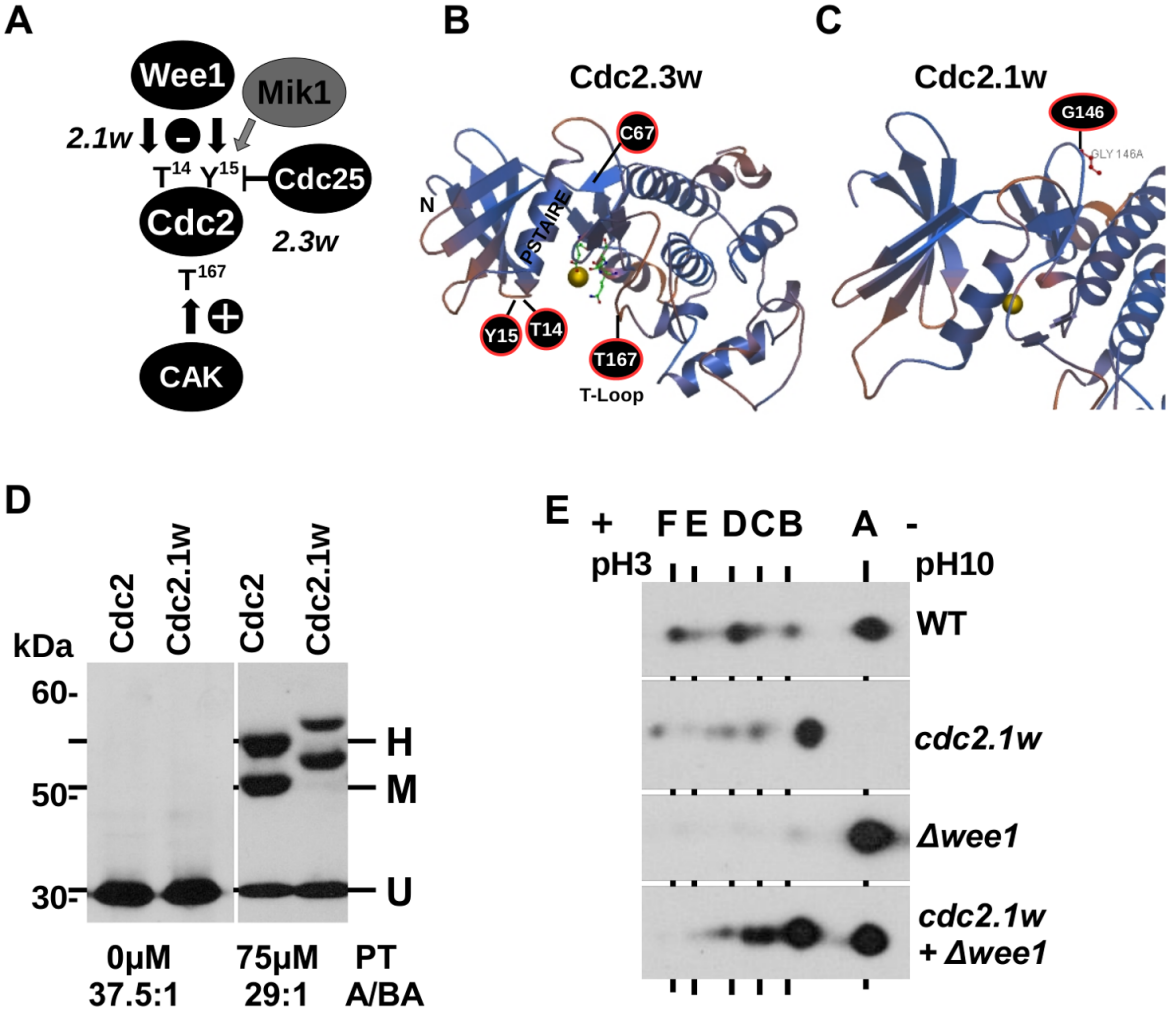
Cdc2 exists in three forms with distinct phosphorylation levels. (A) Phosphorylation of *S*.*pombe* Cdc2 at threonine-14 (T14), tyrosine-15 (Y15) and threonine-167 (T167). Cdc2.1w is insensitive to inhibition by Wee1 kinase, whereas Cdc2.3w does not require activation by Cdc25 phosphatase. (B+C) Model of *S*.*pombe* Cdc2. The model was produced with the Swiss Model tool (template: PDB ID: 4EOM; identity: 66.78%; range: 1-296aa; coverage: 97%). The positions of T14, Y15, C67Y (*cdc2*.*3w*), G146D (*cdc2*.*1w*) and T167 are shown. The ATP ligand and the magnesium ion (yellow) are included. (D) Separation of total protein extracts of the indicated strains on a normal 10% SDS page and a 10% Phos-tag SDS page. The Cdc2 bands H, M and U are indicated. (E) IEF of total protein extracts of the indicated strains on a linear pH gradient (pH3-10).

Since its discovery more than 30 years ago, it remained a mystery how a single point mutation (G146D, Fig 1A and 1C) in *S*.*pombe* Cdc2 (CDK1) can override the cell cycle control by Wee1 kinase [1, 2, 3]. Wee1 phosphorylates the highly conserved tyrosine-15 (Y15) residue at the entrance to the ATP binding site to keep the CDC2-cyclin B complex in its low activity state until cells are ready to enter mitosis [4, 5]. In *S*.*pombe*, Y15 is also modified by Mik1 kinase when cells progress through S phase [6, 7]. Association of Cdc2 with its cyclin partner is stabilised by phosphorylation of T167 in its activation loop (T loop) (T161 in human CDK1; Fig 1B) by the CDK-Activating-Kinase (CAK) Cdc7^Hs^ (Msc6^Sp^, Cak1^Sc^) [8, 9, 10]. Fission yeast cells are unusual as they express two CAK enzymes, with Msc6 being redundant with Csk1 [11]. As modification of T161^Hs^ (T167^Sp^) activates the human CDK1^Cdc2^-cyclin B complex, this phosphorylation is tightly coupled with the inhibitory modification of T14 [12]. Human T14 becomes phosphorylated by Myt1 kinase at the endoplasmic reticulum and Golgi system before the CDK1-cyclin B complex is modified at T161 in the nucleus by the constitutively active CAK [12, 13]. T14 modification levels are much lower in fission yeast compared to mammalian cells and accumulate during S phase in a Wee1-dependent manner [14]. In contrast to human cells, yeast cells possess only one main CDK, Cdc2^Sp^ or Cdc28^Sc^, and in fission yeast an engineered monomolecular Cdc2-cyclin B kinase complex can drive the orderly progression through the cell cycle in the absence of the remaining cyclins [15]. Mitosis is triggered by the abrupt activation of the Cdc2-cyclin B complex upon its dephosphorylation at T14 and Y15 by Cdc25 phosphatase [16, 17]. In human and yeast, activation of the DNA damage checkpoint delays cell cycle progression as Y15 phosphorylation accumulates. This accumulation is caused by the simultaneous removal of Cdc25 from the nucleus and the up-regulation of Wee1 kinase [18, 19, 20, 21].

This model of Cdc2 regulation brings up a second very intriguing question. How can Cdc2 regulate the response to DNA damage, when the Cdc2-cyclin B complex is kept in an inactive state by Y15 phosphorylation? Cdc2 promotes the repair of broken chromosomes by non-homologous end joining (NHEJ) in G1 and by homologous recombination (HR) in G2 by regulating DNA end resection [22, 23, 24]. Interestingly, this regulation is compromised in the *cdc2*.*1w* mutant. The premature rise in Cdc2 activity due to the G146D mutation promotes NHEJ in G2 at the expense of HR by locking the DNA damage checkpoint protein Crb2 (53BP1^Hs^) in its G1 mode [25]. This repair defect may explain why *cdc2*.*1w* cells are only sensitive to DNA replication fork breakage caused by the topoisomerase 1 inhibitor camptothecin (CPT), whereas *Δwee1* cells possess a much broader sensitivity profile [25, 26].

To further understand how Cdc2 exerts its diverse functions, we revisited an important observation which was made more than 25 years ago by Potashkin and Beach (1988) [27]. They reported seven Cdc2 species of differing charge in *S*.*pombe*. Intriguingly, only five species (species: C, D, E, F, G) could be labeled with radioactive orthophosphate while two species (species: A, B) remained unphosphorylated. To assign these species to the known phosphorylation sites T14, Y15 and T167, and to find out which species are the target of the DNA damage checkpoint, we utilized the novel combination of isoelectric focusing (IEF) with Phos-tag SDS electrophoresis (IEFPT) [28]. In the first dimension, IEF separates Cdc2 proteins by their different isoelectric points (IPs). As the IP is equivalent to the overall charge of the protein, it is not only influenced by phosphorylation but also by sulfation of tyrosine, acetylation or methylation of lysine, deamidation of asparagine and glutamine, or carboxylation of glutamate [29]. In the second dimension, Cdc2 species are separated by their degree of phosphorylation as the Phos-tag acrylamide slows down the movement of phosphorylated protein by the formation of a reversible manganese-phosphate-Phos-tag complex [30]. Consistent with the previous report [27], the IEFPT technology revealed seven dominant Cdc2 forms in logarithmically growing wild type cells. Amongst these seven forms, four species are highly phosphorylated (H), one species contains a medium level of phosphorylation (M) and two species are unphosphorylated (U). While all four H-forms bind to cyclin B (Cdc13^Sp^), which implies that they carry the T167 phosphorylation, only two H-forms (He + Hf) are the target of the DNA damage checkpoint as the Y15 modification is restricted to them. The most extensively phosphorylated form, Hf, may also be modified at T14 as its abundance strongly declines in the *cdc2*.*T14A* mutant. The modification status of the M-form is not yet known, but it is noteworthy that it doesn`t have a high affinity for cyclin B. Intriguingly, both unphosphorylted forms (Ua + Ub) associate with the DNA damage checkpoint kinase Chk1 independently of DNA damage. Applying this IEFPT analysis to *cdc2*.*1w*, *Δwee1* and *cdc2*.*1w Δwee1* mutants, we found that Cdc2.1w kinase is aberrantly modified in a manner dependent on Wee1. Taken together, our results entertain a model such that unphosphorylated Cdc2 contributes to the DNA damage response via Chk1 while the Y15 modified phospho-forms regulate cell cycle progression.

## Materials and Methods

### Strains

wild type (*h- ade6-704 leu1-32 ura4-D18*), *cdc2*.*1w* (*h- leu1-32 ura4-D18 cdc2*.*1w*), *cdc13-245 (h- leu1-32 ura4-D18 ade6-704*, *cdc13-245*), *cdc13-245 cdc2*.*1w (h- leu1-32 ura4-D18 ade6-704 cdc13-245 cdc2*.*1w*), *Δwee1* (*h- wee1*::*ura4+ ade6-704 leu1-32 ura4-D18*), c*dc2*.*1w Δwee1* (*cdc2*.*1w wee1*::*ura4+ ade6-704 leu1-32 ura4-D18)*, *Δmik1 (mik1*::*ura4+ ade6-704 leu1-32 ura4-D18)*, *cdc2*.*1w Δmik1 (cdc2*.*1w mik1*::*ura4+ ade6-704 leu1-32 ura4-D18)*, *cdc2*.*T14A (h- cdc2*.*L7*::*cdc2*.*T14A ade6-M216 his3*.*D1 leu1*.*32 ura4*.*D18*), *chk1-HA *[31], *cdc13-HA* (*h- cdc13-HA-ura4* ^*+*^ *leu1*.*32 ura4-D18*).

### Antibodies and Materials

Anti-Cdc2 antibody (ABCAM ab5467); anti-Cdc2-Y15P antibody (Cell Signalling 9111S), HA-probe antibody (F-7) (Santa Cruz, sc-7392), acetylated-lysine antibody (Cell Signaling, 9814S), Phos-tag acrylamide (AAL-107 Wako Ldt), IEF Dry Strips pH3-10 linear (Biorad, 163–2000), anti-HA magnetic beads (Pierce, 88836), protein A agarose (Pierce, IP06).

### Biochemical Techniques

Isoelectric focusing: 5x10^8^ cells were harvested, washed with 1xPBS and resuspended in 300μl IEF buffer (7M urea, 2M thiourea, 4% CHAPS, 50mM DTT, 0.2% carrier ampholytes [pH3-10 linear], 0.0002% bromophenol blue) with six microspoons of zirconia-silica beads (Thistle Scientific) in a 2ml mircotube. Extracts were prepared on a cell disruptor (Scientific Industries). Insoluble material was removed from the supernatant at 12,000rpm for 5min. 10μl of supernatant was added to 115μl of IEF buffer and loaded onto a Ready Dry strip (linear pH3-10). Strips were rehydrated for 12 hours at 50V on a Biorad PROTEAN IEF cell and focused using the rapid ΔV method over 10,000Vh. Strips were sequentially incubated for 10min in 2.0ml of equilibration buffer I (6M urea 0.375M Tris-HCl (pH8.8), 2% SDS, 20% glycerol, 2% DTT) and 2.0ml equilibration buffer II (6M urea, 0.375M Tris-HCl (pH8.8), 2% SDS, 20% Glycerol, 2.5% Iodoacetamide) prior to electrophoresis on a 10% SDS page. Phos-tag SDS acrylamide gels (9%) were prepared according the manufacturer’s instructions with 75μM Phos-tag. Manganese ions were removed prior to the transfer onto nitrocellulose membranes by incubating the gel three-times for 10min in transfer buffer with 20mM EDTA pH8.0. Native Electrophoresis: [32]; Total Protein Extracts: [33], Soluble Protein Extracts: [34].

Immunoprecipitation: 5x10^8^ cells were harvested, washed with 1xPBS and resuspended in 300μl lysis buffer (20mM HEPES pH7.2, 200mM potassium acetate, 5mM β-glycerolphosphate, 1mM NaF, 0.1% Nonidet P-40, 10% glycerol, 1mM EDTA pH 8.0, 2mM activated sodium-orthovanadate, 1:100 Protease Inhibitor Cocktail IV (Melford P2402)). Soluble extracts were prepared as described above but at 4°C. Precipitation was performed overnight with 100μl protein extract, 900μl lysis buffer, 4μl anti-Cdc2 antibody and 30μl protein A agarose, or 20μl anti-HA magnetic beads. The beads were washed five times with 1ml lysis buffer without inhibitors and resuspended in 100μl IEF buffer without DTT. 20μl of the eluted material was added to 100μl IEF buffer and loaded on a IEF strip.

Phosphatase treatment: Cdc2 protein was precipitated with the anti-Cdc2 antibody and the sample was washed three times with lysis buffer and twice with water. Half of the bead sample was incubated in 1x phosphatase buffer only, and the second half in the buffer plus the phosphatase enzyme (5μl Calf Intestinal Alkaline Phosphatase (New England Biolabs, M0290S) or 5μl lambda protein phosphatase (NEB P0753S)). The bead samples were incubated for 60min at 37°C (CIP) or at 30°C (lambda). The reactions were stopped by the addition of 100μl IEF buffer (without DTT). 20μl of the bead samples were subjected to IEF.

## Results

### Cdc2 exists in three pools with different phosphorylation levels

Phospho-amino acid analysis conducted by Potashkin and Beach (1988) [27], and by Gould and Nurse (1989) [5] detected high levels of phosphorylated threonine and tyrosine, but only very little phospho-serine when Cdc2 was affinity purified from logarithmically growing *S*.*pombe* cells. Isoelectric focusing (IEF) of radioactively labeled, affinity-purified Cdc2 protein revealed six dominant forms from which only four are phosphorylated [27]. Later work identified T14, Y15 and T167 as the only three phosphorylation sites in Cdc2^Sp^ modified by Wee1 (T14, Y15) and the two CAK kinases Msc6 and Csk1 (T167), respectively (Fig 1A and 1B) [5, 14, 10, 11]. How these three phosphorylation events map onto the four phospho-species and what the function of the two unphosphorylated forms is remained so far unexplained. Informed by our recent finding that the mutant kinase Cdc2.1w (G146D; Fig 1C) imprints a G1-like DNA repair mode onto G2 cells [25], we wanted to find out whether the phosphorylation pattern of Cdc2.1w differs from the wild type kinase. We addressed this question by a combination of IEF and Phos-tag SDS electrophoresis (IEFPT). The Phos-tag compound forms reversible metal-phosphate complexes thereby slowing down the migration of phosphorylated proteins [30]. The Phos-tag reagent has a very high affinity to phosphate (Kd = 25nM) and a more than 5000 times lower affinity for the related sulfate ion. We started our analysis by separating total protein extracts prepared from logarithmically growing wild type and *cdc2*.*1w* cells on a normal 10% SDS page and on a 10% SDS page containing 75μM Phos-tag. Cdc2 was visualized with an anti-Cdc2 antibody after transfer onto nitrocellulose membrane. While only one 35kDa band (Cdc2: 297aa, 34.36 kDa, IP: 7.68) was detect after separation on a normal 10% SDS page, electrophoresis in the presence of the Phos-tag reagent revealed three bands (H, M, U) (Fig 1D). The two upper bands (H+M) migrated more slowly with an apparent molecular weight of 55kDa and 50kDa, while the third band (U) possessed the expected size of 35kDa. Given that Cdc2 is modified at three sites (T14+Y15+T167), we speculated that the highly modified H band represents fully phosphorylated Cdc2, whereas band M contains the partially modified kinase and band U the unmodified protein. Interestingly, the Cdc2.1w kinase produced a similar pattern, but the mobility of bands H and M was reduced compared to the wild type protein (Fig 1D).

Since this reduction in mobility suggests additional phosphorylation events specific to Cdc2.1w, we subjected total protein extracts to IEF on a linear pH gradient ranging from 3 to 10 prior to electrophoresis on a normal 10% SDS page. This revealed six Cdc2 forms with discrete isoelectric points (IP) in wild type extracts (Fig 1E, panel 1). Since Potashkin and Beach reported a similar IEF pattern [27], we labeled the six forms A to F as in their original publication with F being the most negative (acidic) form and A the least negative (alkaline) form (Fig 1E). The presence of one additional negative charge at position 146 in Cdc2.1w shifted all forms closer to the acidic anode as reported previously by Potashkin and Beach (Fig 1E, panel 2). Given the reduced movement of the Cdc2.1w bands H and M in the presence of the Phos-tag reagent (Fig 1D), aberrant phosphorylation may contribute to this shift in addition to the G146D mutation. Interestingly, loss of Wee1 kinase (*Δwee1*) reduced the intensity of the more negative forms (B to F) while strongly increasing the amount of the least negative form A (Fig 1E, panel 3). A decrease in Cdc2 phosphorylation in the absence of Wee1 is expected as only Mik1 remains to modify T14 and Y15 [6]. We were however surprised by the IEF pattern of the *Δwee1 cdc2*.*1w* extract. The most negative form F was no longer detectable and a strong signal at the position of the least negative A form re-appeared (Fig 1E, panel 4). Since this A form has lost its acidic shift, it is possible that Cdc2.1w (G146D) gains a new post-translational modification in a *wee1* deletion strain which neutralizes the negative charge of D146. Since the intensity of the forms B, C, D and E remained very high in the *Δwee1 cdc2*.*1w* strain, loss of Wee1 may allow another kinase to modify Cdc2.1w. The strongest candidate is Mik1 kinase as it shares an overlapping substrate specificity with Wee1 and because a *Δwee1Δmik1* double mutant dies from mitotic lethality [6].

### Wee1 kinase contributes to the aberrant post-translational modification of Cdc2.1w

**Fig 2:**
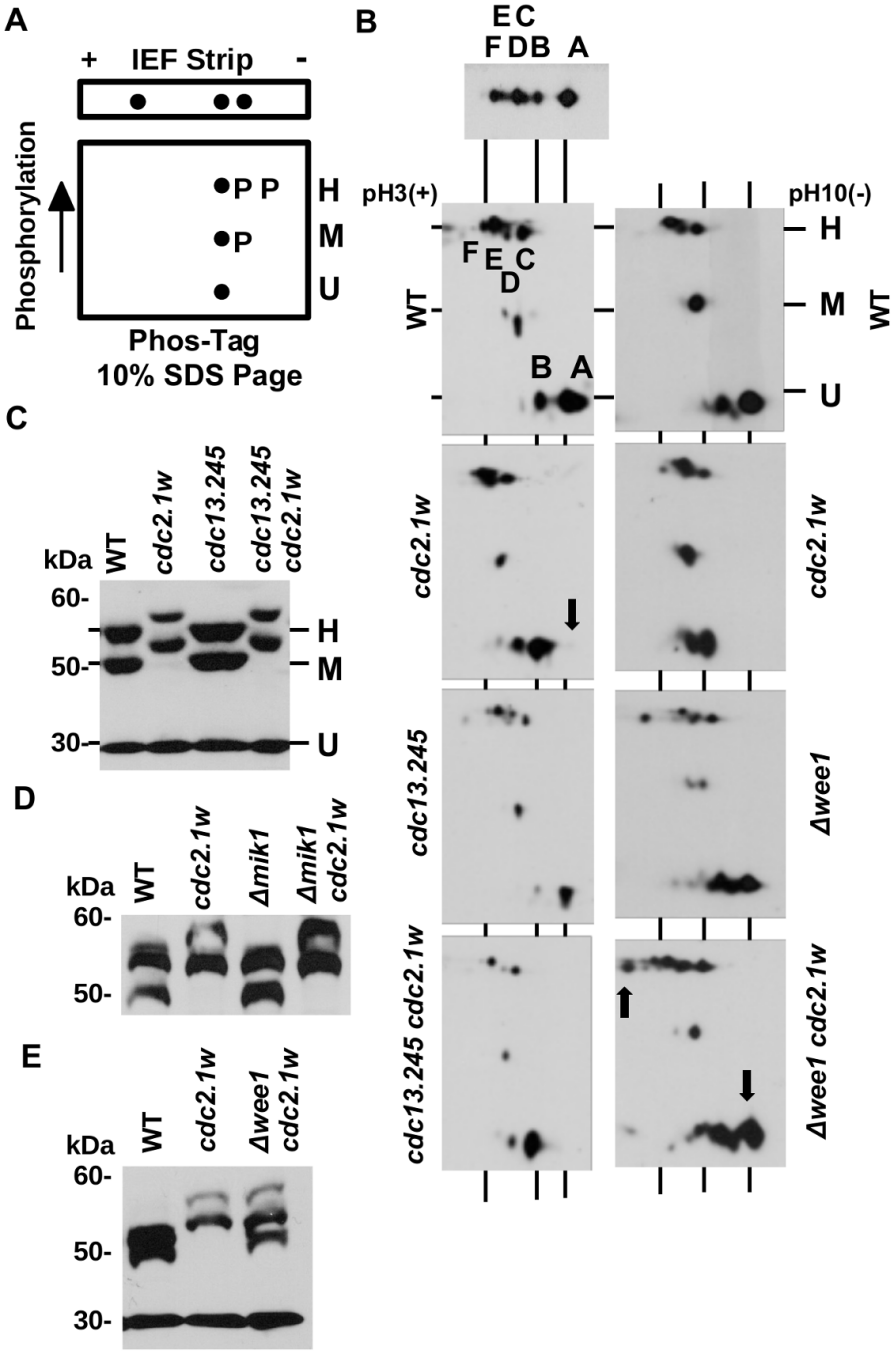
Wee1 kinase contributes to the aberrant modification of Cdc2.1w. (A) IEFPT analysis: IEF strips were placed on top of a 10% Phos-tag SDS page. The IEF strip separates Cdc2 by its isoelectric values and the Phos-tag SDS page by its degree of phosphorylation. (B) IEFPT analysis of total extracts prepared from the indicated strains. The mobility of the Cdc2 bands H, M and U is indicated. The top panel shows an example of the normal IEF pattern of wild type cells. The two wild type and *cdc2*.*1w* experiments were conducted independently. The arrows indicate the acidic shift of forms A and B in the *cdc2*.*1w* mutant, its reversal in the *cdc2*.*1w Δwee1* strain, and the additional, highly phosphorylated species in the H range in the *cdc2*.*1w Δwee1* strain. (C-E) Phos-tag analysis of total protein extracts prepared from the indicated strains.

To correlate the IEF forms A-F with the experiments conducted by Potashkin and Beach, we combined IEF with Phos-tag SDS electrophoresis (IEFPT) as this should confirm the phosphorylation status of these forms (Fig 2A). As expected, the four negative forms C to F migrated more slowly at the height of the H band, whereas the two least negative species (A+B) migrated faster at the level of the unmodified U band (Fig 2B). While these observations are consistent with the previous report [27], we observed one additional spot at the medium phosphorylated M level. This led us to conclude that spots A and B represent the two unphosphorylated Cdc2 species and forms C to F the four phosphorylated forms described by Potashkin and Beach. Since the A and B species have distinct isoelectric values, but are not phosphorylated, other post-translational modifications must contribute to their charge difference. For example, *S*.*cerevisiae* Cdc28^Cdc2^ is acetylated at lysine-40 (K33 in human and *S*.*pombe* Cdc2) which removes one positive charge and is crucial for its function [35]. We probed affinity-purified Cdc2 samples with an antibody that detects lysine residues acetylated at their ε-amino group, but failed to detect a positive signal (not shown). This negative result could however be due to the limited specificity of this antibody.

The IEFPT analysis of a *cdc2*.*1w* extract produced the wild type pattern with one important difference. The forms A and B displayed a significant acidic shift towards the anode which indicates a more negative charge (Fig 2B, panel 2). To test whether this shift is dependent on a mutation in cyclin B (Cdc13) or on Wee1 kinase, we repeated the experiment with a *cdc2*.*1w cdc13-245* double mutant, which carries a temperature-sensitive allele of cyclin B [22], and a *cdc2*.*1w Δwee1* strain. The mutation in cyclin B had a limited impact on the IEFPT pattern at the semi-permissive temperature of 30°C as only the intensity of spots D and F was reduced. Consistent with the earlier IEF experiment, loss of Wee1 resulted in the re-appearance of an unphosphorylated Cdc2 form lacking the acidic shift, and we observed one additional, very negative spot in the H range (Fig 2B, bottom panel). We also run extracts from these strains on a normal 9% SDS page with Phos-tag. While neither the mutation in cyclin B (Fig 2C) nor loss of Mik1 kinase (Fig 2D) affected the mobility of the phosphorylated Cdc2.1w bands M and H, inactivation of Wee1 resulted in the appearance of an additional band with a reduced phosphorylation level (Fig 2E). Taken together, these observations suggest that Wee1 contributes to the aberrant phosphorylation pattern of Cdc2.1w as detected by Phos-tag electrophoresis and protects the mutated kinase from a modification which can neutralize its negative charge at D146 as indicated by the accumulation of unphosphorylated protein in the A-B sector of the IEFPT gel.

### Only two forms of Cdc2 are tyrosine-15 phosphorylated

**Fig 3:**
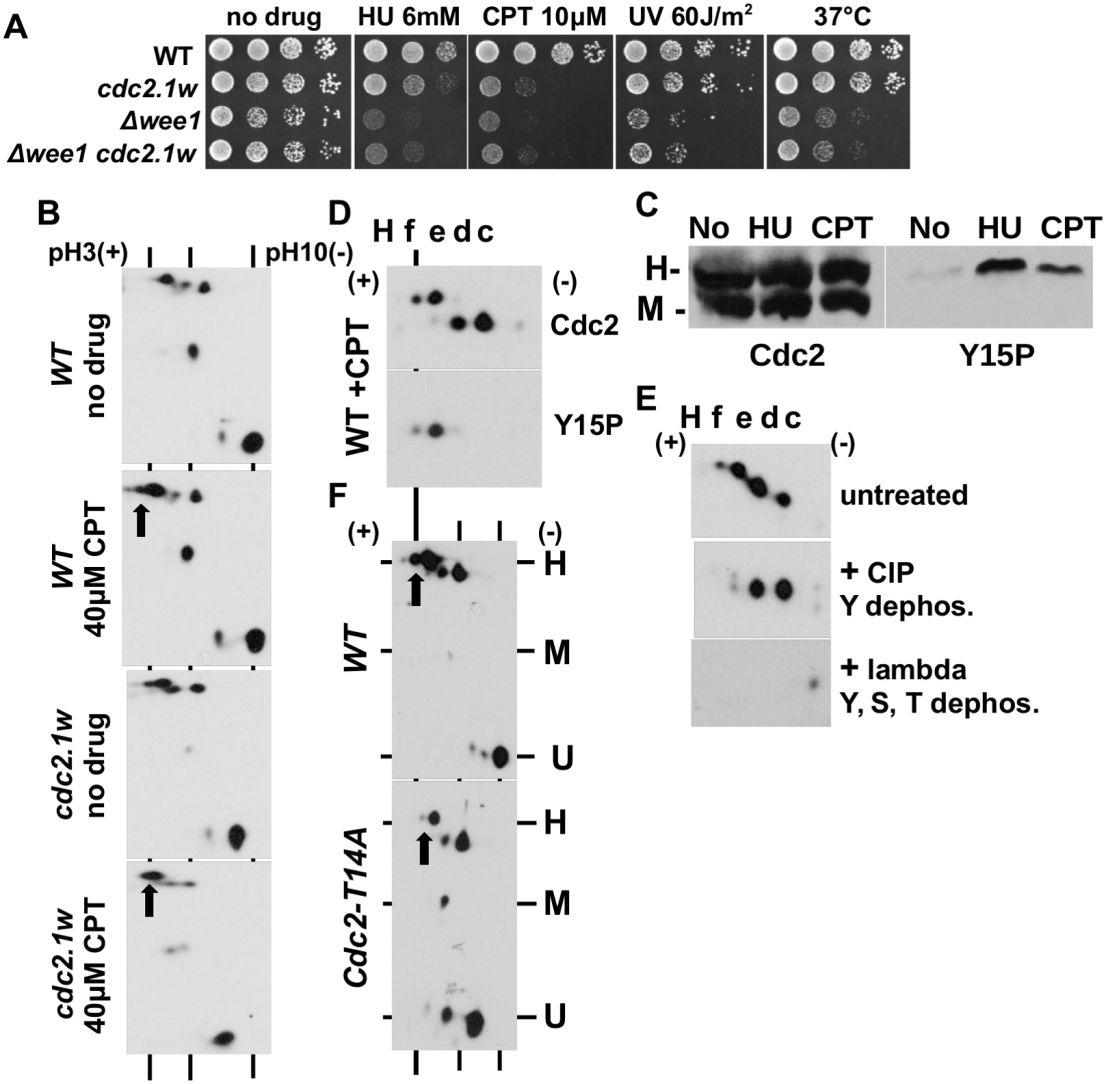
Only Cdc2-He and Cdc2-Hf are phosphorylated at tyrosine 15. (A) Serial dilutions (10-fold; starting with 10^7^ cells/ml) of the listed strains were spotted onto YEA plates containing the indicated drugs. The plates were incubated at 30°C for 3 days. One YEA plate was incubated at 37°C. (B) IEFPT analysis of wild type and *cdc2*.*1w* cells grown in rich medium with 40μM camptothecin (CPT) or without the drug. The arrows indicate the increase in the abundance of the Cdc2 forms He and Hf. (C) Phos-tag analysis of total protein extracts prepared from wild type cells grown in rich medium with 12mM HU (hydroxyurea causes DNA replication arrest), with 40μM CPT (camptothecin causes DNA replication fork damage) or no drug. The total protein extracts were probed with the anti-Cdc2 antibody (left panel) or the anti-Cdc2-Y15 phospho-antibody (right panel). (D) Cdc2 was immunoprecipitated with an anti-Cdc2 antibody from CPT-treated wild type cells. The enriched kinase was either probed with the anti-Cdc2 antibody (top panel) or the anti-Cdc2-Y15 phospho-antibody (bottom panel). Only the H range is shown. (E) Cdc2 was immunoprecipitated with an anti-Cdc2 antibody from CPT-treated wild type cells and treated with Calf Intestinal Alkaline Phosphatase (CIP), which dephosphorylates tyrosine, with lambda protein phosphatase, which dephosphorylates tyrosine, threonine and serine, or left untreated. The protein was detected with the anti-Cdc2 antibody. (F) IEFPT analysis of wild type and *cdc2*.*T14A* cells grown in rich medium without a drug.

The G146D mutation in a loop behind the ATP binding site (Fig 1C) renders *cdc2*.*1w* cells specifically sensitive to the topoisomerase 1 poison camptothecin (CPT) (Fig 3A) [25]. To test whether CPT treatment affects the IEFPT pattern, we grew wild type and *cdc2*.*1w* cells for 4 hours in rich medium without the drug or with 40μM CPT. DNA replication fork damage by CPT induced an increase in the intensity of the forms E and F in wild type extracts suggesting that both species are Y15 phosphorylated. A similar change was evident in the CPT-treated *cdc2*.*1w* extracts (Fig 3B). We also treated wild type cells with 12mM hydroxyurea (HU) (which only stalls replication forks) or 40 μM CPT for 4 hours to test which of the three Phos-tag bands reacts with the anti-Y15P antibody. While untreated cells contained only a low amount of this modification, Y15 phosphorylation accumulated during the incubation with both drugs only in the H band (Fig 3C).

To confirm whether the phospho-forms E and F in the H region become Y15 modified, we immunoprecipitated Cdc2 from wild type extracts treated with 40μM CPT for 4 hours and probed the affinity purified Cdc2 protein with the anti-Cdc2 and the anti-Y15P antibody. While the anti-Cdc2 antibody detected all four phospho-forms (C-F), the anti-Y15P antibody identified only the two most negative species E and F (Fig 3D). This important finding reveals that only two of the seven Cdc2 species (He + Hf) are the target of the DNA damage checkpoint and involved in delaying mitosis in the presence of DNA damage. We confirmed this conclusion by treating the immunoprecipitated wild type Cdc2 protein after CPT incubation with either Calf Intestinal Alkaline Phosphatase (CIP), which preferentially de-phosphorylates tyrosine residues [36], or with lambda protein phosphatase, which removes phosphates from tyrosine, serine and threonine. Consistent with the previous result, CIP only removed the spots E and F, whereas lambda phosphatase removed all four spots (C-F) from the H region (Fig 3E). These results show that Cdc2-He and Cdc2-Hf are phosphoryated at Y15 in addition to threonine residues, while Cdc2-Hc and Cdc2-Hd are only threonine modified. To distinguish between T14 and T167 modifications, we compared the IEFPT pattern of untreated wild type and *cdc2*.*T14A* cells. This identified Cdc2-Hf as a strong candidate for carrying the T14 and the Y15 modifications as the intensity of the F species significantly declined in the *cdc2*.*T14A* mutant. The second Y15 modified form, Cdc2-He, was not affected in the mutant strain (Fig 3F). Unfortunately, a similar experiment with a *cdc2*.*T167A* mutant is not possible as this strain permanently arrests in mitosis [10] and there are no commercial anti-Cdc2-T14P and anti-Cdc2-T167P antibodies which react with the threonine-phosphorylated *S*.*pombe* kinase.

### Cyclin B binds to all highly phosphorylated forms

**Fig 4:**
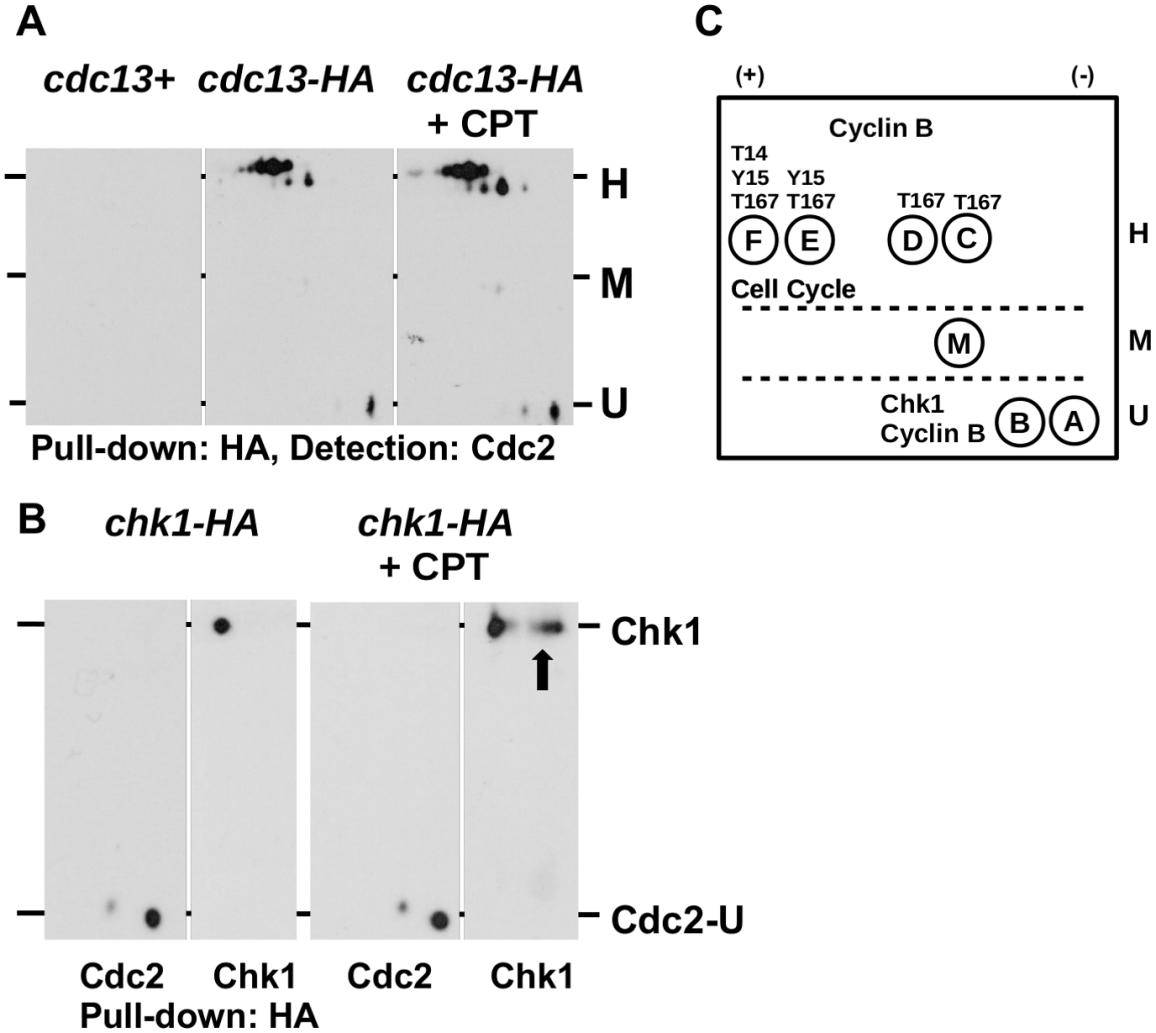
Unphosphorylated Cdc2 associates with Chk1 kinase. (A) Cyclin B (Cdc13) was immunoprecipitated from untreated and CPT-treated (40μM CPT, 4h) *cdc13-HA* cells and the precipitated protein was detected with the anti-Cdc2 antibody. Wild type (*cdc13+*) cells were included as a negative control. (B) The DNA damage checkpoint kinase Chk1 was immunoprecipitated from untreated and CPT-treated (40μM CPT, 4h) *chk1-HA* cells and the precipitated protein was detected with the anti-Cdc2 antibody and an anti-HA antibody (Chk1). The arrow indicates the DNA damage-induced phosphorylation of Chk1. Cdc2-U indicates the unphosphorylated Cdc2 species Ua and Ub. (C) Model. Since all four forms (C-F) in the H range bind to cyclin B (Cdc13) they are likely to be T167 phosphorylated. Cdc2-Hf may carry phosphate residues at T14, Y15 and T157, whereas Cdc2-He may be only phosphorylated at Y15 and T167. Cdc2-He and Cdc2-Hf are both involved in cell cycle regulation. Cdc2-Hc and Cdc2-Hd associate with cyclin B and may therefore be T167 modified. The unphosphorylated forms Cdc2-Ua and Cdc2-Ub associate with Chk1 and Cyclin B and may act in the response to DNA damage. Cdc2-M may be serine phosphorylated.

Since we could not test the T167 phosphorylation directly, we decided to immunoprecipitate cyclin B (Cdc13) from an affinity tagged *cdc13-HA* strain to find out which Cdc2 forms associate with it. This assay indicates T167 phosphorylation as this modification is required for the stable formation of the Cdc2-cyclin B complex [37]. As shown in Fig 4A, Cdc13 associates with all four phospho-forms (C-F) in the H range. We also noted three additional spots in this area which we did not observe when Cdc2 was pulled down with the anti-Cdc2 antibody (Figs 3D and 4A). This implies that not all Cdc2-cyclin B complexes are accessible to the anti-Cdc2 antibody. Interestingly, Cdc13 appears to have a very low affinity to the medium phosphorylated M form as this species was almost absent from the immunopreciptated material (Fig 4A). As reported previously for the human CDK1^CDC2^-cyclin B complex [12], Cdc13 associates also with the unphosphorylated CDK1 kinase. The cellular roles of the unphosphorylated CDK1-cyclin B complex, which resides in the cytoplasm in human cells, is not yet known.

### Unphosphorylated Cdc2 associates with the DNA damage checkpoint kinase Chk1

We reported recently two Cdc2 protein complexes in *S*.*pombe* [25]. A large complex which may contain Cdc2, cyclin B and the DNA damage checkpoint kinase Chk1, and a smaller complex which may contain only Cdc2 and cyclin B. To find out to which Cdc2 species Chk1 binds, we repeated the immunoprecipitation experiment with a *chk1-HA* strain [31]. The cells were treated with either 40μM CPT for 4 hours or left untreated. As shown in Fig 4B, the *chk1-HA* pull down material contained only the two unphosphorylated forms Cdc2-Ua and Cdc2-Ub, but none of the phosphorylated species. Activation of Chk1 by the DNA damage checkpoint in the presence of CPT was evident from the two phosphorylated forms of the kinase which migrate closer to the alkaline end of the strip as reported previously [33]. This unexpected result implies that the unphosphorylated Cdc2 kinase resides in the larger complex with Chk1. This conclusion is also in line with the association of cyclin B (Cdc13) with the Cdc2-U forms (Fig 4A). Since Cdc2 is not phosphorylated in this complex it is unlikely that Cdc2 is the target of Chk1. It is more likely that this complex acts in the response to DNA damage independently of the cell cycle activity of Cdc2 kinase.

## Discussion

The phosphorylation of human and *S*.*pombe* CDK1 (Cdc2) kinase is restricted to the three amino acids T14, Y15 and T161^Hs^ (T167^Sp^) [8, 5, 14, 10, 38]. Earlier work by Potashkin and Beach revealed four phosphorylated forms and two unphosphorylated species of Cdc2 in logarithmically growing *S*.*pombe* cells [27]. How these forms map onto the three phosphorylation sites and how they contribute to the divers roles of Cdc2 in cell cycle regulation and DNA repair remained so far unresolved.

### Cdc2-Hf may act in S phase while Cdc2-He may regulate the G2-M transition

Our first key finding is the observation that only two Cdc2 forms are modified at tyrosine 15 (Y15). This identifies Cdc2-He and Cdc2-Hf as the target of Wee1 and Mik1 kinase in cell cycle regulation (Figs 3 and 4C). Interestingly, both forms differ in the phosphorylation status of threonine 14 (T14) as only the intensity of the Hf species declines in the *cdc2*.*T14A* mutant (Fig 3F). It is therefore possible that Cdc2-Hf regulates S phase progression as the T14 modification peaks during DNA replication in a Wee1-dependent manner [14], whereas Cdc2-Hf coordinates the G2-M transition. The association of both forms with cyclin B (Cdc13) (Fig 4A) strongly implies that Cdc2-Hf and Cdc2-He are also modified at T167. While phosphorylation of the T-loop at T167^Sp^ (T161^Hs^) is not essential for cyclin B binding [8], it significantly stabilises the Cdc2-cyclin B complex [39]. Further support for a model in which Cdc2-Hf regulates S phase and Cdc2-He the onset of mitosis stems from two previously published findings. Only the simultaneous mutation of T14 and Y15 to alanine abolishes the link between the completion of DNA replication and the initiation of mitosis [40], and reactivation of DNA replication origins after initiation of S phase is prevented by a chromatin bound Cdc2-cyclin B complex [41]. It seems that the phosphorylation of T14 adopted a different role in yeast compared to human cells. While the T14 phosphorylation of human CDK1 is tightly coupled to the activating T161 modification to prevent premature activation of the largely cytoplasmic CDK1-cyclin B complex during interphase [12], the same modification may earmark the *S*.*pombe* Cdc2-cyclin B complex for its roles in S phase.

### What are the roles of Cdc2-Hc and Cdc2-Hd?

The absence of the Y15 modification strongly indicates a role for Cdc2-Hc and Cdc2-Hd outside of cell cycle regulation (Fig 3D). Both forms may only be T167 modified since their intensity hardly changes in the *cdc2*.*T14A* mutant (Fig 3F) and because *S*.*pombe* Cdc2 is probably not serine phosphorylated [40]. This conclusion is further supported by their association with cyclin B (Fig 4A). This raises however an interesting question. If both forms are neither T14 nor Y15 modified, why do cells not enter mitosis prematurely? As this is not the case, Cdc2-Hc and Cdc2-Hd may be excluded from the nuclear spindle pole body (centrosome in higher eukaryotic cells) where the cell cycle active forms of Cdc2 reside [42]. Their different isoelectric points (IP) suggest also the presence of additional modifications which may counterbalance the activating T167 phosphorylation. Active *S*.*cerevisiae* Cdc28^Cdc2^ is acetylated [35] which would affect the IP, but we could not detect acetylation at the ε-amino group of lysine residues by probing affinity-purified Cdc2 with a modification specific antibody (not shown).

### Unphosphorylated Cdc2 may play a role in the DNA damage response

Our second key finding is the association of the DNA damage checkpoint kinase Chk1 with the two unphosphorylated forms Cdc2-Ua and Cdc2-Ub (Fig 4B). *S*.*pombe* Chk1 is activated in G1 when cells pass through the start point of S phase [43] and in G2 when DNA damage is detected [44] to prevent premature mitotis. We recently reported the presence of two distinct Cdc2 protein complexes in *S*.*pombe* [25]. Cdc2-Ua and Cdc2-Ub may be part of the larger complex (more than 700kDa) which also contained Chk1, whereas Cdc2-He and Cdc2-Hf may associate in the smaller complex (200-400kDa) from which Chk1 was absent. Cyclin B was present in both complexes which is in line with its association with Cdc2-Ua and Cdc2-Ub (Fig 4A). Interestingly, a similar unphosphorylated CDK1^Cdc2^-cyclin B complex is also present in human cells [12]. Cdc2 is probably not the target of Chk1 in this larger complex as neither Cdc2-Ua nor Cdc2-Ub are phosphorylated (Fig 3). It is more likely that Chk1 uses the unphosphorylated Cdc2-cyclin B complex to gain access to sites in the cell where it can execute its functions in cell cycle regulation and DNA repair. One such site could be a licensed DNA replication origin since Chk1 is activated when cells pass through the start point of S phase. Whether Chk1 is still modified by Rad3^ATR^ kinase in the Cdc2 complex in the response to DNA damage is as yet unclear, but possible. Activation of Chk1 by Rad3 at the G1-S transition is mediated by the chromatin protein Rad4 which also acts at the start of S phase [45], and the Cdc2-cyclin B complex binds to replication start sites [41]. Taken together, this evidence supports a model in which the Cdc2-Chk1 complex regulates early DNA replication events in the presence of DNA damage.

### Is Cdc2.1w aberrantly modified by Wee1 kinase?

As reported previously [27], the presence of the negatively charged aspartate at position 146 (G146D) shifts all Cdc2 forms closer to the positive anode (Fig 1E). Intriguingly, some of this shift is reversed upon the deletion of *wee1* as unphosphorylated Cdc2.1w protein accumulates in the *cdc2*.*1w Δwee1* mutant (Fig 1F). Compared to *Δwee1* cells, the impact of Wee1 inactivation on the abundance of the phosphorylated Cdc2.1w forms C to F is however much reduced in the *cdc2*.*1w Δwee1* double mutant. These findings led to two interesting conclusions. Firstly, the negative charge at position 146 in the Cdc2.1w kinase may be neutralized by a novel post-translational modification in the absence of Wee1 since the acidic shift of the unphosphorylated form is no longer detectable (Figs 1E and 2B). Secondly, another kinase may gain access to Cdc2.1w in *cdc2*.*1w Δwee1* cells as the intensity of the phosphorylated forms remains high (Fig 1E). This kinase is most likely Mik1 given its overlapping substrate specificity with Wee1 [6]. Mik1 may not be able to fully replace Wee1 in the *cdc2*.*1w Δwee1* strain since a faster migrating band (i.e. reduced phosphorylation) appears on the Phos-Tag gel (Fig 2E). On balance, these observations imply that Wee1 shields Cdc2.1w from aberrant modifications by Mik1 and other enzymes.

### Why are Δwee1 cells DNA damage sensitive?

A longstanding mystery is the broad sensitivity profile of *Δwee1* cells (Fig 2A) [25] which is independent of the premature onset of mitosis [26]. So far no direct role of Wee1 in DNA repair has been discovered which points towards an indirect cause of the DNA damage sensitivity. Our findings provide now an explanation. The accumulation of unphosphorylated Cdc2 in *Δwee1* cells (Figs 1E and 2B) may interfere with the DNA damage activities of Cdc2-Ua and Cdc2-Ub thus contributing to the broad sensitivity spectrum. This idea is in line with the change of the sensitivity profile of the *cdc2*.*1w* mutant upon deletion of *wee1*. While *cdc2*.*1w* cells are specifically sensitive to CPT, they adopt the broader profile of *Δwee1* cells upon the loss of the kinase (Fig 3A).

### What are the roles of the medium phosphorylated Cdc2 form?

In addition to the six main Cdc2 forms, *S*.*pombe* cells contain one medium phosphorylated species (Cdc2-M) (Fig 2B). This form does not react with the anti-Y15 phospho-antibody and has a very low affinity for cyclin B (Fig 4A). Its abundance is also independent of the *cdc2*.*T14A* mutation (Fig 3F). Taken together, Cdc2-M is probably not phosphorylated at T14, Y15 or T167. We have however observed that its abundance is lower in the *cdc2*.*1w* mutant (Fig 3C) and that a second species in the M range appears in the absence of Wee1 (Fig 2B). One possibility is that Cdc2-M is phosphorylated at serine residues. Although very little phospho-serine has been found in *S*.*pombe* [10, 27], chicken CDK1 is modified at serine 277 which is also present in *S*.*pombe* Cdc2 [46].

## Conclusion

The outcomes of this work inform a model in which two distinct Cdc2 pools regulate cell cycle progression (Cdc2-He + Cdc2-Hf) and the response to DNA damage (Cdc2-Ua + Cdc2-Ub) (Fig 4C). Further work is however required to understand how Cdc2-Ua and Cdc2-Ub impact on the DNA damage response, and which post-translational modifications define their distinct isoelectric points. It would also be important to identify the cellular activities of Cdc2-Hc and Cdc2-Hd, which associate with cyclin B but lack the Y15 phosphorylation.
